# Impact of Japan’s 2024 Physician Work Style Reform on Pediatricians’ Working Hours and Associated Factors

**DOI:** 10.3390/healthcare13151815

**Published:** 2025-07-25

**Authors:** Masatoshi Ishikawa, Ryoma Seto, Michiko Oguro, Yoshino Sato

**Affiliations:** Research Institute, Tokyo Healthcare University, Higashi Gotanda 4-1-17, Shinagawa, Tokyo 141-8648, Japan

**Keywords:** pediatrician, working hours, Japan, work reform

## Abstract

**Background/Objectives:** Long working hours among pediatricians negatively affect their health and patient safety. In Japan, the Ministry of Health, Labour and Welfare launched the “Work Style Reform for Physicians” in 2024. However, whether these reforms have effectively reduced pediatricians’ working hours remains unclear. We surveyed pediatricians and pediatric residents working in hospital pediatric departments to assess whether the reform has reduced their long working hours. **Methods:** A questionnaire was distributed to pediatricians in hospitals, collecting data on demographics, working hours, night shifts, and other working conditions. A multivariate logistic regression analysis identified factors associated with working ≥60 and ≥80 h on a weekly basis. **Results:** Questionnaires were sent to 835 hospitals, with valid responses from 815 pediatricians across 316 hospitals. Among them, 31.7% worked 50–60 h per week, 18.4% worked 60–70 h, 7.7% worked 70–80 h, and 4.9% worked >80 h. Factors associated with working >60 h included being <30 years old and working in a department with five or more physicians. Pediatricians working >80 h were more likely to have a cardiology subspecialty and work in a department with five or more physicians. **Conclusions:** Although the “Work Style Reform for Physicians” has reduced long working hours among pediatricians, many still experience excessive workloads.

## 1. Introduction

Long working hours among doctors adversely affect their health and compromise patient safety, making this a global public health concern [1,2,3]. Pediatricians in Japan report some of the longest working hours among physicians in OECD (Organisation for Economic Co-operation and Development) countries, particularly when compared with their counterparts in the United States and European Union member states [4].

To address this issue, in 2003, the Accreditation Council for Graduate Medical Education (ACGME) in the United States imposed a limit of <80 h per week on residents’ working hours. This decision was based on evidence indicating that excessive working hours adversely affect residents’ health and increase the risk of medical errors [5,6].

In Japan, no comparable restrictions have been implemented for pediatricians. However, in response to concerns about physician overwork, Japan’s Ministry of Health, Labour and Welfare established the *Study Group on the Work Style Reform for Doctors* to explore potential solutions. As part of this initiative, it was announced that, starting in April 2024, the annual upper limit for overtime hours for residents would be set at 1860 h, aligning with the ACGME standards in the United States [7].

A nationwide survey conducted in Japan in 2020 highlighted the prevalence of long working hours among pediatricians, with 51.7% working more than 60 h per week and 14.4% exceeding 80 h weekly [8]. Since then, the Ministry of Health, Labour and Welfare has promoted the “Work Style Reform for Physicians.” Although several studies have described physician overwork in Japan, no empirical evaluation has assessed whether the April 2024 Work Style Reform for Physicians has actually reduced pediatricians’ working hours, despite its positioning as a high-priority policy intervention. This study, thus, fills a critical gap by quantifying post-reform changes and identifying the remaining predictors of overwork.

International evidence, such as the 80-hour weekly cap set by the ACGME in the United States, links work hour limits to reduced medical errors and improved physician well-being. By analogy, Japan’s 2024 reform warrants a focused impact evaluation in regard to the pediatrician workforce. This study aimed to assess changes in pediatricians’ working hours and to identify background factors contributing to overwork, based on the following questions:

(1) Have long working hours among pediatricians improved following the 2024 workstyle reform?
(2) After implementation of the 2024 reform, which physician- and hospital-level factors remain significantly associated with working hours exceeding 60 h per week, and how do these differ from pre-reform predictors?

We designed a cross-sectional survey to measure pediatricians’ working hours and perceptions before and after the April 2024 reform. Our analysis plan included descriptive statistics, chi-square tests to carry out Likert comparisons, and multivariable logistic regression to identify predictors of overwork.

## 2. Materials and Methods

### 2.1. Participants

This study targeted 835 pediatric hospitals, whose names are publicly available through the Hospital Bed Function Reporting System [9]. In June 2024, a survey request was sent to the heads of the pediatric departments at these hospitals, and a web-based questionnaire was conducted. Pediatric departments were selected based on prior findings indicating a high prevalence of overwork among pediatricians [8]. The survey was administered via a secure online platform (Google Forms) in June 2024. No financial compensation was provided to participants. In this study, “residents” are defined as physicians taking part in postgraduate pediatric specialty training programs, typically within 2–6 years of graduating from medical school.

This study utilized a cross-sectional design, based on an anonymous, web-based survey. The target population comprised pediatricians and pediatric residents working in hospital pediatric departments across Japan. Responses were excluded if information on the respondent’s working hours or hospital affiliation was missing.

Questionnaires were distributed to 835 hospitals, and valid responses were obtained from 815 pediatricians across 316 hospitals, resulting in a response rate of 37.8%.

Respondents’ characteristics were described overall and for those with weekly working hours of ≥60 h and ≥80 h. The respondents’ characteristics included their sex, age, job title, subspecialty, marital status, number of children, annual income (including part-time income), weekly working hours, number of night shifts per month, work schedule after night shifts, the hospital’s foundational entity, total number of beds, regional classification, and number of full-time pediatricians (Table 1).

Age was categorized into five groups: <30 years, 30s, 40s, 50s, and ≥60 years. Job titles were classified into four groups: department head, staff, resident, and other. Subspecialties were categorized into eight groups: neonatology, neurology, allergology, cardiology, hematology, endocrinology, nephrology, and others. Annual income, including part-time earnings, was divided into four categories: CNY <6 million yen, CNY 6–10 million, 10–14 million, and CNY ≥14 million. Overtime hours were categorized into six groups: <40 h, 40–50 h, 50–60 h, 60–70 h, 70–80 h, and ≥80 h. The number of night shifts per month was classified into three categories: <5, 5–9, and ≥10 shifts. Work schedules following a night shift were categorized into five groups: no night shift, regular shift (full-day shift), half-day off (work until noon), full-day off (work until morning), and others. The foundational entities of the hospitals were classified into four groups: public/government, national university, private university, and private. The total number of beds was categorized into five groups: <200, 200–400, 400–600, 600–800, and >800 beds. The regional characteristics were classified into three categories, namely urban, intermediate, and rural, based on a combination of population size and population density in 344 secondary medical care areas [10]. The questionnaire was developed by the authors in consultation with two senior pediatricians and pilot tested with 20 physicians. All items are original; the test–retest reliability of the pilot data yielded a Cronbach’s α of 0.82.

Next, we summarized the responses regarding how the participants perceived their working hours (very short, somewhat short, somewhat long, very long, none of the above); whether they felt their working environment, including reduced working hours, had improved compared with conditions in 2019 (before the COVID-19 pandemic) (significantly worse, somewhat worse, unknown, somewhat improved, significantly improved, none of the above); and their perception of appropriate weekly working hours (<40 h, 40–49 h, 50–59 h, 60–69 h, 70–79 h, or ≥80 h) (Table 2). Likert scale items were summarized using frequency distributions and compared via chi-square tests to assess the perceived changes in working hours before versus after the reform (see Table 2).

The response rate was 37.8%, comparable to prior national physician surveys in Japan (30–40%), suggesting reasonable sample representativeness. The categories for ‘appropriate work hours per week’ follow international benchmarks, including the ACGME and European Society and Japanese practice guidelines.

Thereafter, the responses regarding whether night/day shift permissions had been obtained (already obtained, to be obtained in the future, no plans to obtain, none of the above), the average nap duration during night shifts (<2 h, 2–4 h, 4–6 h, >6 h, none of the above), and how the average workload during night shifts compared with the definition of a night shift (very little, somewhat little, same as day shift, somewhat excessive, very excessive, none of the above) were assessed (Table 3).

“Night/day shift permission” refers to formal approval granted by hospital administration allowing physicians to perform overnight duties, as required by labor regulations for scheduling and overtime management. The definition of night shift includes the following:

- Involves mild or short-term tasks that do not require special measures;
- Rarely entails duties similar to daytime responsibilities.

Examples include the following:

- During an on-call ward shift, responding to changes in the condition of a few high-risk patients by conducting interviews and examinations and providing instructions and confirmations to nurses and other staff members;
- On holidays or nights when outpatient visits are not typically expected (e.g., non-rotation days), managing a small number of mildly ill outpatients or fluctuations in the condition of regular patients, including conducting interviews and examinations and providing instructions and confirmations to nurses and other staff members.

### 2.2. Statistical Analyses

To examine the relationship between pediatricians’ working hours and background factors, a multivariable logistic regression analysis was performed, using weekly working hours of ≥60 h or ≥80 h as the dependent variables. Respondent attributes, including their sex, age, job title, subspecialty, marital status, number of children, annual income (including part-time income), weekly working hours, number of night shifts per month, work schedule following night shifts, hospital foundational entity, total number of hospital beds, regional classification, and number of full-time pediatricians, were included as independent variables (Table 4).

For the statistical analyses, *p*-values <0.05 were considered statistically significant. All the statistical analyses were performed using STATA 17.0. StataCorp. 2021. Stata: Release 17. College Station, TX, USA: StataCorp LLC.

### 2.3. Ethical Considerations

This study was conducted with the approval of the Tokyo Healthcare University Research Ethics Committee for Human Studies (Approval Number: Kyo-023-24B). The study’s purpose and measures for ensuring secure data management were stated on the first page of the questionnaire. Participants were informed that their involvement was entirely voluntary. The results were analyzed separately from the respondent’s personal information to ensure anonymity.

## 3. Results

Questionnaires were distributed to 835 hospitals, and valid responses were obtained from 815 pediatricians across 316 hospitals (response rate: 37.8%). These 815 pediatricians accounted for 7.4% of the 11,030 hospital-based pediatricians, as reported in the 2022 Ministry of Health, Labour and Welfare statistics on physicians, dentists, and pharmacists [11].

As shown in Table 1, 34.4% of the respondents were women, and the largest age group was 40–49 years, comprising 31.0% of the participants. Regarding marital status, 80.2% of the respondents were married. The most common subspecialty was neurology (12.8%), whereas the most frequently reported income bracket was CNY ≥14 million. The most common weekly working hours were 50–59 h (31.7%), with 31.0% reporting ≥60 h/week and 4.9% reporting ≥80 h/week. Regarding night shifts, 50.6% of the respondents reported working 5–9 shifts per month. With respect to hospital characteristics, 63.1% of the respondents worked at public hospitals, and 53.3% were employed in intermediate cities. The most frequently reported hospital size was 400–600 beds.

Regarding the perceptions of their working hours, 46.1% of the respondents considered their hours “just right,” whereas 40.0% found them to be “somewhat long” and 8.6% found them to be “very long.” Compared with 2019, the most common response regarding changes in the work environment was “unknown” (39.0%), followed by “somewhat improved” (32.0%). When asked about appropriate weekly working hours, 48.0% selected “40–50 h,” followed by 29.2%, who chose “50–60 h.”

In regard to night shifts, 53.4% of the respondents had “already obtained” permission, whereas 12.6% had “no plans to obtain” permission. The most common nap duration during night shifts was “2–4 h” (32.5%), followed by “4–6 h” (30.3%). Regarding the workload during night shifts, the most frequent response was “somewhat excessive” (26.9%), followed by “very excessive” (20.9%) and “same as day shift” (20.6%).

The results of the multivariate logistic regression analysis are presented in Table 4. When overtime hours of ≥60 h was used as the dependent variable, significant associations were observed for the following factors: age ≥60 years (reference: <30 years) (odds ratio [OR]: 0.27; 95% confidence interval [CI]: 0.08–0.82; *p* = 0.02), 5–9 pediatricians in the department (reference: <5) (OR: 2.04; 95% CI: 1.17–3.54; *p* = 0.01), and ≥15 pediatricians in the department (reference: <5) (OR: 2.45; 95% CI: 1.20–5.03; *p* = 0.01).

When overtime hours of ≥80 h was used as the dependent variable, significant associations were identified for the department size: 5–9 pediatricians (reference: <5) (OR: 13.35; 95% CI: 1.49–119.77; *p* = 0.02), 10–14 pediatricians (reference: <5) (OR: 11.93; 95% CI: 1.12–127.23; *p* = 0.04), and ≥15 pediatricians (reference: <5) (OR: 12.81; 95% CI: 1.15–142.39; *p* = 0.04). Although not statistically significant, a subspecialty in cardiology (reference: neonatology) showed a potential association (OR: 4.49; 95% CI: 0.94–21.49; *p* = 0.06).

## 4. Discussion

This study revealed that 31.0% of pediatricians worked ≥60 h per week, highlighting the persistence of severe overwork conditions among pediatricians in Japan. These findings can be compared with those of a 2020 national survey, in which 51.7% of hospital-based pediatricians worked ≥60 h per week and 14.4% worked ≥80 h per week [8]. Our results, showing reductions to 31.0% and 4.9%, respectively, suggest a downward trend in extreme overwork since the implementation of the reform. Because the 2020 survey and our 2024 data were collected under different contexts (pre- vs. post-pandemic and pre- vs. post-reform), direct comparisons should be interpreted with caution.

Prolonged working hours (≥60 h per week) are strongly associated with an increased risk of mental disorders resulting from psychological stress and cardiovascular diseases. This threshold is commonly referred to as the “karoshi” (death from overwork) level and serves as the standard for recognizing work-related accidents [11,12]. Based on the findings of this study, 31.0% of respondents were working under conditions that exceeded this threshold.

A comparison with a prior survey on pediatricians’ working hours conducted in 2020 revealed that the proportions of those working ≥60 h per week and ≥80 h per week were 31.0% (compared with 51.7% in 2020) and 4.9% (compared with 14.4% in 2020), respectively [8]. These findings indicate an overall reduction in working hours.

Under Japan’s “Work Style Reform for Physicians” initiative, spearheaded by the Ministry of Health, Labour and Welfare, new regulations will be implemented from April 2024, capping annual overtime at 960 h for most physicians. However, an exception has been made for residents, allowing up to 1860 h [7]. This threshold translates to an average of >80 h per week. Based on the findings of this study, 4.9% of respondents may have exceeded this limit. Consequently, prolonged working hours will become illegal after April 2024, necessitating urgent measures to reduce excessive workloads.

Among physicians working 60–80 h per week, a significant association was observed with the number of pediatricians working at their hospital. In Japan, the large number of hospitals relative to the population has led to a dispersion of medical resources, resulting in a lack of centralization and an inefficient healthcare delivery system [12]. In hospitals with relatively few physicians, efforts to maintain functionality with a limited workforce likely include measures such as reducing nighttime outpatient services, focusing on mild cases, and restricting the scope of medical services. To effectively reduce physician working hours, reforms to the healthcare delivery system, including the centralization of medical resources, are necessary. Notably, larger departments, despite having more staff, may paradoxically experience less flexible scheduling or higher expectations for coverage per physician. Institutional culture and subspecialty demands may also contribute to workload imbalances, highlighting the need for workload redistribution policies and task redesign at the departmental level. Centralization refers to the consolidation of medical functions into fewer, better-resourced institutions. In Japan, the excessive dispersion of pediatric services across many small facilities leads to inefficiency and staffing shortages. Centralizing care may help reduce individual physician workloads by enabling more efficient allocation of duties.

Being aged <30 years was identified as a significant factor associated with working ≥60 h per week. Physicians in this age group are typically residents who have completed their initial postgraduate training and are working toward obtaining a specialist pediatric certification. These residents, known as specialty trainees, are required to acquire extensive knowledge and technical skills, making long working hours a global issue [13]. In the United States, the ACGME has implemented working hour restrictions for residents, with reports indicating no significant differences in patient outcomes or resident satisfaction compared with non-restricted groups [14,15].

The ORs suggest that working in larger departments (≥15 pediatricians) is significantly associated with overwork. This may indicate that such departments, although better staffed overall, impose more complex schedules or demand greater subspecialized care. These findings highlight the need to reassess duty allocation systems, even in seemingly well-resourced settings.

Although not statistically significant, this study observed an association between cardiology as a subspecialty and working >80 h per week, suggesting that working hours may vary by specialty. In Japan, specialty and subspecialty choices are left to individual physicians and can be made either upon graduation from medical school or after obtaining a medical license [12]. Consequently, physicians may avoid selecting demanding subspecialties or may transition to less demanding ones [16].

Previous studies have reported significant associations between long working hours and factors such as sex, not having children, an annual income of CNY >16 million, and employment at a private university hospital. However, in this study, no significant associations were observed, even after accounting for similar factors [8]. One possible explanation is that variations in the implementation of the physician work style reform among individuals and hospitals may have influenced the results.

As shown in Table 2, although many pediatricians still perceived their working hours as long, most reported that their situation had either remained the same or somewhat improved compared with that of around 2019. Additionally, most physicians expressed a preference for working hours below the “karoshi” threshold. Several measures can be considered to reduce excessive working hours, including task shifting to other healthcare professionals, the use of information technology to improve efficiency, and addressing the dispersion and imbalance of resources [7,16]. Although hospital management plays a critical role in these efforts, political support, such as specialized training in task shifting, may further facilitate these measures.

These findings raise concerns that the ease with which healthcare institutions obtain “night shift permissions” may lead to inaccurate recording of working hours. In other words, although reported working hours may appear to have decreased on paper, actual workloads may not have been reduced. In hospitals with night shift duty approval, if “regular tasks” are performed during night shifts, those hours should be classified as work hours, with appropriate wages, including necessary overtime pay, provided separately from the night shift allowance [17].

If night shift duties are treated as working hours, annual overtime limits could be exceeded, posing challenges for hospitals, particularly in regions facing physician shortages. In such areas, hospitals may have no choice but to obtain night shift duty approval to prevent the collapse of local healthcare systems [18,19]. Given the impact on regional healthcare, it is crucial to improve the working conditions of pediatricians experiencing excessive workloads.

Our findings are consistent with previous studies in Japan and other OECD countries that demonstrate the persistence of long working hours among physicians, despite regulatory reforms. However, our data show a more modest proportion of overwork compared to previous national surveys, suggesting some initial success of the reform. Nonetheless, the continued prevalence of >60 h workweeks among approximately one-third of respondents indicates structural and cultural inertia within medical institutions.

In addition to the United States, several other countries have introduced working hour regulations for physicians, including pediatricians, to safeguard healthcare professionals and patients. In the European Union, the Working Time Directive (Directive 2003/88/EC) limits average weekly working hours to 48 h, including on-call time, across all medical specialties [20]. The United Kingdom strictly enforces this directive, implementing rota systems with mandatory rest periods between shifts and capping weekly hours [21]. Similarly, Germany and France apply comparable restrictions, albeit with negotiated flexibility in certain sectors [22]. In contrast, Japan’s current limit of 1860 h per year applies only to overtime, excluding standard working hours, meaning that total working hours can still exceed 60–80 h per week. These discrepancies highlight that Japan’s regulatory stance remains relatively permissive; further convergence with international standards may be warranted.

A 2023 survey by the Ministry of Health, Labour and Welfare showed that pediatricians remained among the most overworked specialists in Japan, with over 25% reporting ≥60 h workweeks, despite upcoming reforms [23]. According to a 2024 survey by the Japan Medical Association, approximately one-third of hospital physicians reported exceeding the 60 h weekly threshold, particularly in pediatrics and emergency care [24].

### 4.1. Policy Implications and Recommendations

Based on our finding that 4.9% of respondents still work ≥80 h per week, we recommend introducing a comprehensive cap on total working hours, including night and on-call shifts, to prevent health hazards and ensure compliance with new legal standards. Our findings suggest that while the “Work Style Reform for Physicians” has contributed to a reduction in extreme overwork, many pediatricians continue to exceed the threshold considered hazardous to health. Therefore, we propose the following policy recommendations:

First, actual total working hours, including those during night shifts, should be clearly defined and systematically monitored, as underestimation may result from the use of night shift permissions [17]. Second, task-shifting initiatives should be accelerated through targeted training and regulatory reform to enable nurses and allied health professionals to share clinical responsibilities [7,16]. Third, regional healthcare delivery should be restructured by consolidating under-resourced pediatric departments to reduce fragmentation and ensure more equitable workload distribution [12]. We recommend implementing a comprehensive cap on total working hours, including standard, night, and on-call duties, drawing on the European Union model, which integrates all forms of work into a unified limit [20]. Furthermore, compensation schemes should accurately reflect actual workloads by separately recording and remunerating night duties that involve clinical activities equivalent to those performed during daytime shifts [17].

Task shifting should include transferring routine clinical tasks, such as history taking and documentation, to nurses or physician assistants. The use of information technologies, such as electronic medical records and automated triage tools, can also improve efficiency. To address regional disparities, financial and staffing incentives may be necessary to support overburdened hospitals in rural areas.

Such strategies would promote physician well-being, reduce burnout, and help sustain high-quality pediatric care in the face of a diminishing physician workforce.

### 4.2. Study Limitations

This study has some limitations. First, as participation in the survey was voluntary, selection bias may have been present. However, questionnaires were distributed to 835 hospitals, and valid responses were obtained from 815 pediatricians across 316 hospitals, yielding a response rate of 37.8%. This rate is comparable to those reported in prior national physician surveys in Japan, which have typically ranged from 30% to 40% response rates [23,24], suggesting reasonable representativeness of the sample. Although self-reported data are subject to recall and reporting biases, we minimized variability by employing standardized, closed-ended response categories. Similar methodologies are used in national-level physician surveys in Japan and internationally [25,26].

Second, because the survey was self-administered, various information biases may have occurred. For instance, reported weekly working hours may have been inaccurate as a result of the absence of detailed time-tracking studies, potentially leading to misreporting. To mitigate this risk, the questionnaire used consistent and standardized wording, with predefined response categories for working hours, shift patterns, and other variables. This approach aimed to reduce variation in interpretation and improve data reliability across diverse hospital settings.

Additionally, as survey participation was voluntary, definitions of specialized terms were not explicitly provided to avoid influencing the response rate.

Third, although statistical associations between long working hours and background factors were examined, causal relationships could not be established. Unmeasured confounding factors may have influenced the results. For example, it remains unclear whether long working hours are primarily driven by individual choices, institutional policies, or broader structural constraints.

The subgroup of respondents who reported working ≥80 h per week was relatively small (n = 40), limiting the statistical power of related analyses. Future studies should increase sample sizes or employ multi-year data pooling to verify these findings. Future research should utilize time-tracking systems, longitudinal study designs, and in-depth qualitative interviews to better understand the structural and behavioral drivers of overwork.

## 5. Conclusions

This nationwide study revealed that the 2024 “Work Style Reform for Physicians” was associated with a reduction in extreme overwork among hospital-based pediatricians in Japan. Compared to the 2020 national survey, the proportion of pediatricians working ≥60 h/week decreased from 51.7% to 31.0%, and those working ≥80 h/week declined from 14.4% to 4.9%. These trends suggest early signs of improvement following the regulatory intervention.

However, nearly one-third of pediatricians continue to exceed the “karoshi” threshold of 60 h/week, a level linked to elevated risks of cardiovascular disease and mental health disorders. Notably, pediatricians in departments with ≥15 physicians were significantly more likely to report ≥80 h/week, underscoring the paradox of overwork in larger, seemingly better-resourced institutions. Additionally, younger physicians (<30 years) were disproportionately affected, likely due to training requirements and cultural expectations.

To ensure the success of future reforms, comprehensive working hour caps should include night and on-call duties, and night shift approvals must not obscure actual work hours. Broader structural changes, including task shifting, information technology adoption, and pediatric care centralization, are urgently needed. International comparisons suggest that Japan’s current regulatory framework remains lenient; its harmonization with EU standards (48 h/week) may offer long-term benefits for patient safety and physician well-being.

In summary, while Japan’s 2024 reform is a meaningful step forward, persistent overwork among pediatricians highlights the need for continuous policy refinement, monitoring, and systemic support.

## Figures and Tables

**Table 1:** Demographic and professional characteristics of participants.

Category	Total	≥60 h/week	≥80 h/week
**Total number of participants**, *n*	815	253	40
**% of all participants**	100.0%	31.0%	4.9%
**Sex**, ***n*, %**			
Female	34.4%	30.4%	40.0%
Male	65.6%	69.6%	60.0%
**Age**, ***n*, %**			
<30	9.4%	12.3%	15.0%
30–39	29.2%	31.6%	37.5%
40–49	31.0%	31.6%	25.0%
50–59	21.0%	19.8%	20.0%
≥60	9.3%	4.7%	2.5%
**Job title, *n*, %**			
Department head	28.5%	25.3%	20.0%
Staff	52.6%	52.6%	55.0%
Resident	16.8%	20.9%	20.0%
Others	2.1%	1.2%	5.0%
**Subspecialty, *n*, %**			
Neonatology	9.7%	9.1%	7.5%
Neurology	12.8%	10.7%	5.0%
Allergology	10.2%	9.1%	10.0%
Cardiology	9.7%	12.3%	17.5%
Hematology	8.6%	9.5%	7.5%
Endocrinology	8.3%	6.3%	2.5%
Nephrology	5.3%	4.7%	5.0%
Others	35.5%	38.3%	45.0%
**Marital status, *n*, %**			
Yes	80.2%	73.9%	65.0%
No	19.8%	26.1%	35.0%
**Number of children, *n*, %**			
0	31.9%	39.5%	52.5%
1	18.0%	14.2%	10.0%
2	29.9%	28.1%	25.0%
3	16.8%	14.6%	10.0%
≥4	3.3%	3.6%	2.5%
**Annual income (including part-time income), *n*, %**			
<6 million	2.7%	2.0%	2.5%
6–10 million	19.1%	16.2%	20.0%
10–14 million	35.6%	37.2%	40.0%
≥14 million	42.6%	44.7%	37.5%
**Working hours per week, *n*, %**			
<40	7.5%	0.0%	0.0%
40–50	29.8%	0.0%	0.0%
50–60	31.7%	0.0%	0.0%
60–70	18.4%	59.3%	0.0%
70–80	7.7%	24.9%	0.0%
≥80	4.9%	15.8%	100.0%
**Number of night shifts per month, *n*, %**			
<5	26.4%	17.0%	12.5%
5–9	50.6%	47.0%	50.0%
≥10	23.1%	36.0%	37.5%
**Work schedule after night shift, *n*, %**			
No night shifts	17.8%	9.9%	10.0%
Regular shift (full-day shift)	20.7%	28.5%	37.5%
Half-day off (work until noon)	40.4%	40.3%	35.0%
Full-day off (work until morning)	17.7%	18.2%	17.5%
None of the above	3.4%	3.2%	0.0%
**Entity of employer, *n*, %**			
Public	63.1%	59.3%	55.0%
National university	13.0%	15.4%	15.0%
Private university	5.9%	9.1%	15.0%
Private	18.0%	16.2%	15.0%
**Employer’s total number of beds, n (%)**			
<200	5.2%	2.0%	2.5%
≥200–<400	28.2%	26.1%	17.5%
≥400–<600 beds	31.0%	32.0%	30.0%
≥600–<800	21.6%	23.3%	25.0%
≥800	14.0%	16.6%	25.0%
**Area, *n*, %**			
Urban	41.0%	44.7%	42.5%
Intermediate	53.3%	50.2%	55.0%
Rural	5.8%	5.1%	2.5%
**Number of pediatricians, *n*, %**			
<5	19.9%	11.9%	2.5%
5–9	36.1%	38.7%	42.5%
10–14	17.3%	15.8%	17.5%
≥15	26.7%	33.6%	37.5%

**Table 2:** Perceptions of working hours and changes since 2019.

	Total	≥60 h/week	≥80 h/week
**Total number of participants, *n***	815	253	40
**% of all participants**	100.0%	31.0%	4.9%
**Own work time**			
Very short	0.6%	5.1%	0.0%
Somewhat short	3.3%	11.9%	0.0%
Just right	46.1%	39.9%	12.5%
Somewhat long	40.0%	28.9%	50.0%
Very long	8.6%	2.4%	37.5%
None of the above	1.3%	11.9%	0.0%
**Did the work environment improve compared with 2019?**			
Significantly worse	3.4%	0.0%	12.5%
Somewhat worse	8.3%	1.2%	15.0%
Unknown	39.0%	24.9%	40.0%
Somewhat improved	32.0%	54.2%	20.0%
Significantly improved	5.2%	19.4%	2.5%
None of the above	12.0%	0.4%	10.0%
**Appropriate work hours per week**			
<40	10.2%	1.6%	2.5%
40–50	48.0%	27.7%	17.5%
50–60	29.2%	38.3%	17.5%
60–70	8.2%	20.2%	27.5%
70–80	3.1%	8.7%	15.0%
≥80	1.3%	3.6%	20.0%

**Table 3:** Night shift work patterns and perceived workload.

	Total	≥60 h/week	≥80 h/week
**Total number of participants, *n***	815	253	40
**% of all participants**	100.0%	31.0%	4.9%
**Permission for night/day shifts**			
Already obtained	53.4%	58.1%	60.0%
To be obtained in the future	1.8%	0.8%	0.0%
No plans to obtain	12.6%	13.8%	17.5%
None of the above	32.1%	27.3%	22.5%
**Average nap time during the night shift**			
<2 h	7.0%	7.9%	7.5%
2–4 h	32.5%	40.7%	45.0%
4–6 h	30.3%	31.2%	32.5%
≥6 h	5.5%	4.7%	2.5%
None of the above	24.7%	15.4%	12.5%
**Average duties during the night shift**			
Very little	2.2%	1.2%	2.5%
Somewhat little	8.5%	6.7%	10.0%
Same as day shift	20.6%	17.0%	5.0%
Somewhat excessive	26.9%	36.8%	32.5%
Very excessive	20.9%	26.1%	45.0%
None of the above	21.0%	12.3%	5.0%

**Table 4:** Logistic regression analysis of factors associated with longer working hours.

	Over 60 h per Week			Over 80 h per Week		
	OR	95% CI	*p*-Value	OR	95% CI	*p*-Value
**Sex**						
Female	Reference			Reference		
Male	1.08	0.73–1.59	0.71	0.72	0.33–1.60	0.42
**Age**						
<30 years old	Reference			Reference		
30s	0.87	0.42–1.80	0.71	0.81	0.20–3.32	0.77
40s	0.75	0.33–1.72	0.50	0.50	0.09–2.70	0.42
50s	0.64	0.26–1.57	0.33	0.52	0.09–3.19	0.48
60 and older	0.27	0.08–0.82	0.02	0.21	0.01–3.33	0.27
**Job title**						
Department head	Reference			Reference		
Staff	0.79	0.48–1.29	0.35	0.72	0.23–2.18	0.56
Resident	0.86	0.39–1.90	0.72	0.29	0.05–1.65	0.16
Others	0.34	0.07–1.57	0.17	1.86	0.19–18.53	0.60
**Subspecialty**						
Neonatology	Reference			Reference		
Neurology	0.86	0.42–1.77	0.68	0.63	0.09–4.37	0.64
Allergology	1.05	0.50–2.24	0.89	1.99	0.38–10.37	0.42
Cardiology	1.70	0.81–3.55	0.16	4.49	0.94–21.49	0.06
Hematology	1.38	0.64–2.99	0.41	1.50	0.25–8.80	0.66
Endocrinology	0.77	0.34–1.76	0.54	0.41	0.04–4.55	0.47
Nephrology	1.11	0.46–2.68	0.82	1.78	0.25–12.64	0.56
Others	1.38	0.73–2.60	0.32	2.88	0.70–11.95	0.14
**Marital status**						
No	Reference			Reference		
Yes	1.46	0.87–2.49	0.15	1.39	0.49–3.90	0.53
**Number of children**						
0	Reference			Reference		
1	0.65	0.36–1.18	0.16	0.36	0.09–1.41	0.14
2	0.89	0.51–1.57	0.69	0.69	0.21–2.26	0.55
3	0.73	0.38–1.41	0.35	0.42	0.10–1.89	0.26
4 or more	0.96	0.36–2.61	0.94	0.68	0.06–7.14	0.75
**Annual income (including part-time income)**						
<6 million	Reference			Reference		
6–10 million	1.19	0.37–3.84	0.77	0.89	0.09–8.60	0.92
10–14 million	2.20	0.68–7.15	0.19	1.64	0.17–15.91	0.67
<14 million	2.87	0.84–9.79	0.09	1.59	0.15–17.35	0.70
**Number of night shifts per month**						
<5	Reference			Reference		
5–9	0.82	0.42–1.60	0.56	2.10	0.23–18.76	0.51
10 or more	1.89	0.93–3.85	0.08	2.84	0.30–26.59	0.36
**Work schedule after a night shift**						
No night shifts	Reference			Reference		
Regular shift (full-day shift)	1.93	0.83–4.49	0.13	0.78	0.07–9.20	0.84
Half-day off (work until noon)	1.17	0.54–2.54	0.68	0.35	0.03–3.65	0.38
Full-day off (work until morning)	1.42	0.62–3.25	0.41	0.44	0.04–4.99	0.51
None of the above	1.43	0.46–4.45	0.54	1.00		
**Foundational Entity of Employer**						
Public	Reference			Reference		
National university	1.48	0.69–3.18	0.31	0.73	0.15–3.56	0.69
Private university	1.89	0.78–4.58	0.16	2.32	0.44–12.34	0.32
Private	1.40	0.87–2.24	0.16	1.11	0.37–3.34	0.85
**Employer’s Total No. of Beds**						
<200 beds	Reference			Reference		
≥200–<400 beds	2.18	0.75–6.37	0.15	0.49	0.05–5.19	0.55
≥400–<600 beds	2.41	0.82–7.06	0.11	0.62	0.06–6.34	0.69
≥600–<800 beds	1.70	0.55–5.28	0.36	0.70	0.06–7.71	0.77
≥800 beds	1.44	0.43–4.84	0.56	0.86	0.07–10.36	0.90
**Workplace**						
Urban	Reference			Reference		
Intermediate	0.75	0.52–1.09	0.13	1.54	0.69–3.44	0.29
Rural	1.04	0.46–2.35	0.92	1.77	0.17–18.27	0.63
**Number of Pediatricians**						
<5	Reference			Reference		
5–9	2.04	1.17–3.54	0.01 *	13.35	1.49–119.77	0.02 *
10–14	1.75	0.88–3.47	0.11	11.93	1.12–127.23	0.04 *
15 or more	2.45	1.20–5.03	0.01 *	12.81	1.15–142.39	0.04 *

* *p* < 0.05.

## Data Availability

The data presented in this study are available on request from the corresponding author. The data are not publicly available due to ethical restrictions.

## Abbreviations

The following abbreviations are used in this manuscript:

ACGME	Accreditation Council for Graduate Medical Education
CI	confidence interval
OR	odds ratio
